# Association between perioperative factors and the occurrence of postoperative cerebellar mutism syndrome in children

**DOI:** 10.3389/fneur.2026.1862663

**Published:** 2026-09-23

**Authors:** Stefanie Ott, Matthias Hans Belau, Martin Mynarek, Matthias Gelderbloom, Fabian Flottmann, Pedram Emami, Hanno Sebastian Meyer, Lasse Dührsen, Friederike Sophie Groth

**Affiliations:** 1 Department of Neurosurgery, University Medical Center Hamburg-Eppendorf, Hamburg, Germany; 2 Institute of Medical Biometry and Epidemiology, University Medical Center Hamburg-Eppendorf, Hamburg, Germany; 3 Department of Pediatric Hematology and Oncology, University Medical Center Hamburg-Eppendorf, Hamburg, Germany; 4 Department of Neurology, University Medical Center Hamburg-Eppendorf, Hamburg, Germany; 5 Department of Neuroradiology, University Medical Center Hamburg-Eppendorf, Hamburg, Germany

**Keywords:** infratentorial tumor, pediatric brain tumor, pediatric cerebellar mutism, pediatric neurosurgery, postoperative cerebellar mutism

## Abstract

**Purpose:**

Postoperative cerebellar mutism syndrome (pCMS) is a complex condition involving neurological impairment including mutism, hypotonia and dysphagia, that occurs after resection of infratentorial lesions predominantly in children. While the literature has reported several preoperative risk factors, there is little knowledge on the surgeon’s role and perioperative factors. Thus, we aimed to examine the association between perioperative features and pCMS.

**Methods:**

This single-center study included 83 patients who underwent microsurgical resection of a cerebellar lesion in 2013–2022. Controls were matched to cases based on age using coarsened exact matching. There were 20 patients with pCMS. The parameters assessed included age, gender, the histology of the lesion, and the modified Rotterdam Risk Score. Intra- and perioperative factors such as the surgical approach; the extent of resection; the administration of dexamethasone, blood products, coagulative factors, and mannitol; the use of a preoperative cerebrospinal fluid (CSF) drain; and postoperative sodium and hemoglobin levels were examined.

**Results:**

Crude-effect analysis showed associations between pCMS and medulloblastoma histology (odds ratio [OR] = 7.40, 95% confidence interval [CI] = [1.75, 31.17]) and the administration of blood products (OR = 4.82, 95% CI = [1.15, 20.16]). Moreover, there was a significant association between the occurrence of pCMS and hemoglobin (OR = 0.55, CI = [0.31, 0.96]) and sodium (OR = 1.21, 95% CI = [1.00, 1.46]) levels on postoperative day 1. Multivariate analysis confirmed the association with medulloblastoma histology (OR = 4.66, 95% CI = [1.13, 19.15]).

**Conclusion:**

In multivariate analysis, histology was the only significant factor for the development of pCMS. Nonetheless, there was a trend suggesting that the administration of blood products and the postoperative hemoglobin and sodium levels could represent risk factors for development of pCMS in this explorative analysis.

## Introduction

Postoperative cerebellar mutism syndrome (pCMS) is a well-known, complex set of symptoms that develops following neurosurgical resection of infratentorial lesions in pediatric patients. It is characterized by mutism, emotional lability, hypotonia, and dysphagia, with a delayed onset, characteristics in line with the Iceland Delphi consensus (1–4). It may frequently be accompanied by cerebellar motor syndrome, cerebellar cognitive affective syndrome, and brain stem dysfunction, including long tract signs and cranial neuropathies. Some symptoms regress over time, but recovery can be prolonged or even incomplete, especially regarding motor symptoms (1). Residual impairment persists in up to 75% of cases after 2 years of follow-up, and half of the patients present complex dysarthria after 10 years (5). Given the marked improved survival in pediatric neuro-oncology, the effect of postoperative morbidity on later quality of life has become a growing concern (1, 4, 5).

Several studies have defined risk factors for the development of pCMS in pediatric patients. These include a midline location of the lesion, the size of the lesion, infiltration of the brainstem, the dentate nucleus or the superior cerebellar peduncle, and medulloblastoma histology (2, 4–7). Suspected histology of astrocytoma or cerebellar hemispheric location has even been described as protective factor (7). These factors were therefore implemented in preoperative risk stratification models via neuroimaging studies (7, 8). However, researchers have yet to establish significant associations between the development of pCMS and pre-resection surgical hydrocephalus treatment, prone position, usage of ultrasonic aspirator, telovelar approach, vermian splitting, and complete or near total resection (9–11). A recent review on pre and intraoperativ factors could demonstrate an association of pCMS development with vermian incision and gross total resection in terms of changeable intraoperative features (12).

Regarding the perioperative course, Pols et al. (4) showed a 0.5 °C increase in body temperature during the first four postoperative days in children with medulloblastoma. However, there were no significant differences between pre- and postoperative hemoglobin levels in multivariate analysis (4). The authors further suggest that pediatric patients may be more vulnerable to hypovolemia, with hyperthermia potentially representing a manifestation of systemic inflammatory response syndrome (SIRS) in association with pCMS (4). Another group has discussed a so-called “two hit” theory and operation-associated injury (13).

Some authors have adopted a more neurofunctional perspective. Cerebellar mutism is partially regarded as a supratentorial disorder (5) or as a consequence of secondary diaschisis affecting the cerebellar efferent system (4, 6, 10, 14). Wibroe et al. (15) further compared pCMS with supplementary motor area (SMA) syndrome in adult patients with disrupted interhemispheric connectivity. Cerebello-cerebral circuit dysfunction occurs in both SMA and pCMS syndrome, leading to similarities in clinical presentation, natural course and having a possible surgical trigger (16).

To date, several approaches have sought to integrate mostly preoperative radiological features to estimate the risk of developing pCMS. In 2018, Lui et al. (8) published a magnetic resonance imaging (MRI) model for risk prediction. It was subsequently validated and transformed into the Rotterdam pCMS prediction model in 2020 for preoperative neuroimaging-based risk assessment (7). This model enables straightforward calculation of the Rotterdam Risk Score based on predictors identified on preoperative MRI. By adding imaging factors like compression of cerebellum and brainstem, compression of the upper brainstem and brainstem invasion in sagittal and longitudinal planes, accuracy of a preoperative MR-based risk model could be improved (7).

The objective of this retrospective, single-center study was to evaluate the impact of neurosurgical management and associated perioperative factors on the development of pCMS in children following resection of an infratentorial intra-axial lesion. To enhance the reliability of the results, the Rotterdam pCMS prediction model was applied to preoperative cranial MRI scans.

## Methods

### Study population and design

A retrospective chart review was performed on pediatric patients with space-occupying lesions in the cerebellum who required surgery between January 2013 and January 2022. Only the first operation performed and documented at the University Medical Center Hamburg-Eppendorf (UKE) was included in the dataset. Repeat operations were not considered. Data were obtained from the local documentation systems myMedis KIS (Getinge) and Soarian® Clinicals (Cerner) at the University Medical Center Hamburg-Eppendorf. Images were viewed via GE Zero Footprint (ZFP). Cases were identified with pCMS postoperatively by the clinicians on the postoperative intensive care unit or the peripheral ward. This study included patients for whom information on age (in months), date of surgery, and pCMS status was available. A case–control design was employed, with patients diagnosed with pCMS serving as cases and age-matched patients without pCMS serving as controls. Matching was performed using coarsened exact matching (17).

### Outcome

Daily clinical assessment was conducted following the surgical procedure, initially in the intensive care unit and subsequently in the pediatric ward. The clinician diagnosed pCMS based on various clinical indicators, including diminished speech up to mutism, hypotonia and irritability. The severity of the condition was not evaluated, the duration of the pCMS symptoms was documented.

### Potential predictors

As potential predictors, we examined intraoperative features that, in our opinion, depend at least partly on the surgeon who makes the decision to use them or who has a major influence. These include the administration of dexamethasone (perioperative and during surgery), coagulation factors (tranexamic acid and fresh frozen plasma), blood products (erythrocyte and platelet concentrates), mannitol (during surgery), the use of perioperative cerebrospinal fluid (CSF) drainage (either by external ventricular drainage or by ventriculocisternostomy). Dexamethasone was administered perioperatively to relieve initial symptoms prior to surgery or intraoperatively in case of swelling according to the surgeon’s impression.

We recorded the histology of the operated lesion (tumor type) as well as the duration of the surgery (in minutes), extent of resection and the surgical approach (suboccipital or telovelar). The telovelar approach is distinguished from suboccipital approach by the necessity for opening the fourth ventricle by the surgeon itself, no vermian split has been conducted. Concerning postoperative course the hemoglobin and sodium levels were measured on postoperative day 1, using the reference ranges of the University Medical Center Hamburg-Eppendorf laboratory. Age (months at diagnosis) and sex (female, male) were included as covariables in the multivariate analyses.

### Imaging and neuroradiological assessment

Preoperative imaging was available in all cases. We evaluated the Rotterdam pCMS prediction model retrospectively on children who developed pCMS and with a control group. Evaluation was based on the preoperative MRI, which was partly conducted in the assigning clinic. There was no standard protocol for external imaging.

MRI examinations in the operating hospital were conducted using a 3.0-Tesla Ingenia system. The standard protocol for pediatric brain tumor imaging at our institution adheres to the guidelines proposed by the European Organisation for Research and Treatment of Cancer and the National Brain Tumor Society (18), encompassing three-dimensional (3D) T1-weighted turbo-field-echo (T1-TFE), fluid-attenuated inversion recovery (FLAIR), diffusion-weighted imaging (DWI), and T2-weighted turbo-spin-echo (T2-TSE), executed in both the axial and coronal orientations. Furthermore, the 3D T1-TFE sequence is repeated 3 min after the administration of a gadolinium-based contrast agent (Dotagraf®, 0.2 mL/kg body weight; Bayer, Leverkusen, Germany). Two physicians evaluated each case according to the Rotterdam pCMS prediction model used an axial MP-Rage post-contrast enhanced image and an axial T2 image under neuroradiological assistance. The extent of resection was evaluated by neuroradiological assessment on a regular MRI scan within the first two postoperative days.

### Statistical analysis

Descriptive statistics were used to identify patient characteristics. The coarsened exact matching method was employed to address imbalances between cases and controls, with the coarsening parameter set to age months at diagnosis, equivalent to age years. The matching process was then executed by initially arranging all observations into strata with identical values for age year at diagnosis. Subsequently, all observations within a stratum that lacked at least one observation for each unique value of the case–control indicator variable were eliminated.

Several potential predictors had missing values, most of which were plausibly missing at random within the eligible cohort. Rather than restricting the analysis to complete cases, missing values were handled using multiple imputation by chained equations (30 imputed datasets). Binary predictors (administration of blood products, coagulation factors, mannitol, perioperative dexamethasone, extent of resection, reoperation) were imputed using augmented logistic regression to account for sparse cells; continuous variables (hemoglobin and sodium on the first postoperative day, and the Rotterdam score) were imputed using predictive mean matching. All imputation models included pCMS status, age at diagnosis, and sex as predictors. Coarsened exact matching was performed separately within each imputed dataset, as matching depended only on age at diagnosis, which was fully observed and therefore identical across imputations.

Multiple logistic regression with weights assigned to the stratum of observations was used to compare pCMS status with potential predictors adjusted for the matching variable, and estimates were combined across the 30 imputed datasets using Rubin’s rules. Potential predictors that demonstrated statistical significance in the bivariate analysis were subsequently employed as inputs in multivariable logistic regression analyses. Adjustments were made for the matching variable and sex. The analysis yielded odds ratios (ORs) and 95% confidence intervals (CIs), with a significance level set at *p* < 0.05; given the exploratory nature of the analysis and the number of predictors examined, no formal correction for multiple comparisons was applied, and results should be interpreted as hypothesis-generating. As a complete-case sensitivity analysis, all multivariable models were repeated without imputation; results are reported in Supplementary Table S1. All analyses were performed using STATA MP version 18.

## Results

A total of 127 pediatric patients who underwent surgery for a cerebellar lesion during the study period were screened for eligibility. Of these, 44 patients were excluded because information on age at diagnosis or date of surgery was not available, leaving 83 eligible patients who were entered into coarsened exact matching on age at diagnosis. All 83 eligible patients were retained after matching, with no observations excluded during the matching procedure itself; pCMS occurred in 20 patients, and 63 patients served as age-matched controls. Table 1 shows the characteristics of the study population.

**Table 1:** Characteristics of the study population.

Characteristic	*n*	%	*n*	%	OR^†^	95% CI lower	Upper
	pCMS: No (*n* = 63)		pCMS: Yes (*n* = 20)				
Age, months at diagnosis							
Mean (SD)	86.9 (63.3)		101.1 (60.4)		**–**		
**Sex**							
Male	38	60.3	11	55.0	1.00		
Female	25	39.7	9	45.0	1.02	0.36	2.90

pCMS, posterior cerebellar mutism syndrome; OR, odds ratio; CI, confidence interval; ^†^Odds ratios adjusted for the matching variable (age at diagnosis, months), weighted by coarsened exact matching weights, and pooled across 30 imputed datasets (multiple imputation by chained equations) using Rubin’s rules.

The crude-effect analysis of the perioperative variables revealed associations between the occurrence of pCMS and the hemoglobin (OR = 0.55, 95% CI = [0.31, 0.96]) and sodium (OR = 1.21, 95% CI = [1.00, 1.46]) levels on postoperative day 1 and the administration of blood products (erythrocyte and platelet concentrates) (OR = 4.82, 95% CI = [1.15, 20.16]) (Table 2). No significant differences between the case and control groups according to the Rotterdam Risk Score could be demonstrated (Table 2). Therefore, preoperative risk stratification using this modified score was not beneficial or robust in our cohort.

**Table 2:** Crude-effect analysis of perioperative variables.

Characteristic	*n*	%	*n*	%	OR^†^	95% CI lower	Upper
	pCMS: No (*n* = 63)		pCMS: Yes (*n* = 20)				
Perioperative dexamethasone							
No	14	22.2	7	35.0	1.00		
Yes	40	63.5	11	55.0	0.38	0.11	1.30
Missing	9	14.3	2	10.0			
Dexamethasone during surgery							
No	54	85.7	15	75.0	1.00		
Yes	7	11.1	5	25.0	4.23	0.94	19.03
Missing	2	3.2	0	0.0			
Mannitol during surgery							
No	58	92.1	16	80.0	1.00		
Yes	3	4.8	4	20.0	4.23	0.81	22.03
Missing	2	3.2	0	0.0			
EC or PC during surgery (blood products)							
No	55	87.3	14	70.0	1.00		
Yes	6	9.5	6	30.0	4.82	1.15	20.16
Missing	2	3.2	0	0.0			
TXA or FFP/coagulation factors							
No	53	84.1	15	75.0	1.00		
Yes	8	12.7	5	25.0	3.30	0.79	13.79
Missing	2	3.2	0	0.0			
Rotterdam score							
Low (0–49 points)	12	19.0	0	0.0			
Medium (50–99 points)	19	30.2	5	25.0	1.00		
High (100–145 points)	32	50.8	15	75.0	1.07′	0.30	3.76
Hemoglobin, day 1 post-surgery (g/dL)							
Mean (SD)	10.06 (1.25)		9.51 (1.02)		0.55	0.31	0.96
Sodium, day 1 post-surgery (mmol/L)							
Mean (SD)	139.74 (4.20)		141.45 (3.27)		1.21	1.00	1.46

CMS, cerebellar mutism syndrome; OR, odds ratio; CI, confidence interval; EC, erythrocyte concentrates; PC, platelet concentrates; TXA, tranexamic acid; FFP, fresh frozen plasma;† Odds ratios adjusted for the matching variable (age at diagnosis, months), weighted by coarsened exact matching weights, and pooled across 30 imputed datasets (multiple imputation by chained equations) using Rubin’s rules.′ Rotterdam score High vs. Low/Medium combined (Low and Medium could not be modeled as separate reference categories because the Low category contains no pCMS cases across most imputed datasets, causing unstable/varying omitted-category sets).

Among the surgical characteristics, there were associations between the occurrence of pCMS and medulloblastoma histology (OR = 7.40, 95% CI = [1.75, 31.17]) (Table 3). Regarding histology, astrocytomas were predominantly pilocytic, with only two cases classified as anaplastic astrocytomas.

**Table 3:** Crude-effect analysis of surgical characteristics.

Characteristic	*n*	%	*n*	%	OR†	95% CI lower	Upper
	pCMS: No (*n* = 63)		pCMS: Yes (*n* = 20)				
Suboccipital surgical approach							
No	5	7.9	1	5.0	1.00		
Yes	53	84.1	18	90.0	1.36	0.13	14.85
Missing	5	7.9	1	5.0			
Telovelar surgical approach							
No	48	76.2	11	55.0	1.00		
Yes	10	15.9	8	40.0	1.86	0.61	5.70
Missing	5	7.9	1	5.0			
Extent of resection							
Complete	28	44.4	12	60.0	1.00		
Subtotal	28	44.4	8	40.0	0.81	0.28	2.38
Missing	7	11.1	0	0.0			
Tumor type							
Astrocytoma	29	46.0	5	25.0	1.00		
Ependymoma	10	15.9	2	10.0	0.60	0.10	3.51
Medulloblastoma	11	17.5	10	50.0	7.40	1.75	31.17
ATRT	5	7.9	2	10.0	2.92	0.33	25.93
Haemangioblastoma	1	1.6	0	0.0			
Other	7	11.1	1	5.0	1.00	0.09	11.54
Repeat operation (any, ever)							
No	40	63.5	15	75.0	1.00		
Yes	22	34.9	5	25.0	0.48	0.15	1.53
Missing	1	1.6	0	0.0			

CMS, cerebellar mutism syndrome; OR, odds ratio; CI, confidence interval; ^†^Odds ratios adjusted for the matching variable (age at diagnosis, months), weighted by coarsened exact matching weights, and pooled across 30 imputed datasets (multiple imputation by chained equations) using Rubin’s rules. Haemangioblastoma: OR not estimable (*n* = 1, no pCMS cases).

In the next step, we included the variables that reached significance in bivariate analysis in stepwise forward multivariate modeling, resulting in a final model with four variables. Models 1–4, reflecting the included factors, are presented in Table 4 and graphically illustrated in Figure 1. There was an association between the occurrence of pCMS and medulloblastoma histology (OR = 4.66, 95% CI = [1.13, 19.15]). The relatively small number of cases (n = 20) contributed to wide confidence intervals, although the 1:4 case-to-control ratio provided reasonable statistical power.

**Table 4:** Associations between relevant exposure variables and post-operative pediatric CMS (multiple imputation, *m* = 30, weighted by coarsened exact matching).

Model 1				Model 2			Model 3			Model 4		
OR‡		95% CI Lower	Upper	OR‡	95% CI Lower	Upper	OR‡	95% CI Lower	Upper	OR‡	95% CI Lower	Upper
Medulloblastoma												
No	1.00			1.00			1.00			1.00		
Yes	7.86	2.19	28.17	6.39	1.71	23.80	5.16	1.28	20.86	4.66	1.13	19.15
EC or PC during surgery												
No				1.00			1.00			1.00		
Yes				3.05	0.63	14.76	2.73	0.54	13.96	2.69	0.50	14.48
Hemoglobin, day 1 post-surgery (g/dL)												
							0.76	0.40	1.45	0.80	0.42	1.53
Sodium, day 1 post-surgery (mmol/L)												
										1.11	0.90	1.36

OR, odds ratio; CI, confidence interval; EC, erythrocyte concentrates; PC, platelet concentrates; mmol/L, millimoles per liter; g/dL, gram per deciliter. ^‡^Adjusted for the matching variable (age at diagnosis, months) and sex, weighted by coarsened exact matching weights, and pooled across 30 imputed datasets (multiple imputation by chained equations) using Rubin’s rules. Repeat operation was evaluated but not included in the final models: in bivariate analysis it was not significantly associated with pCMS (OR = 0.48, 95% CI 0.15–1.53) once correctly defined as “any reoperation during follow-up” (rather than “this operation was itself a reoperation”).

**Figure 1:**
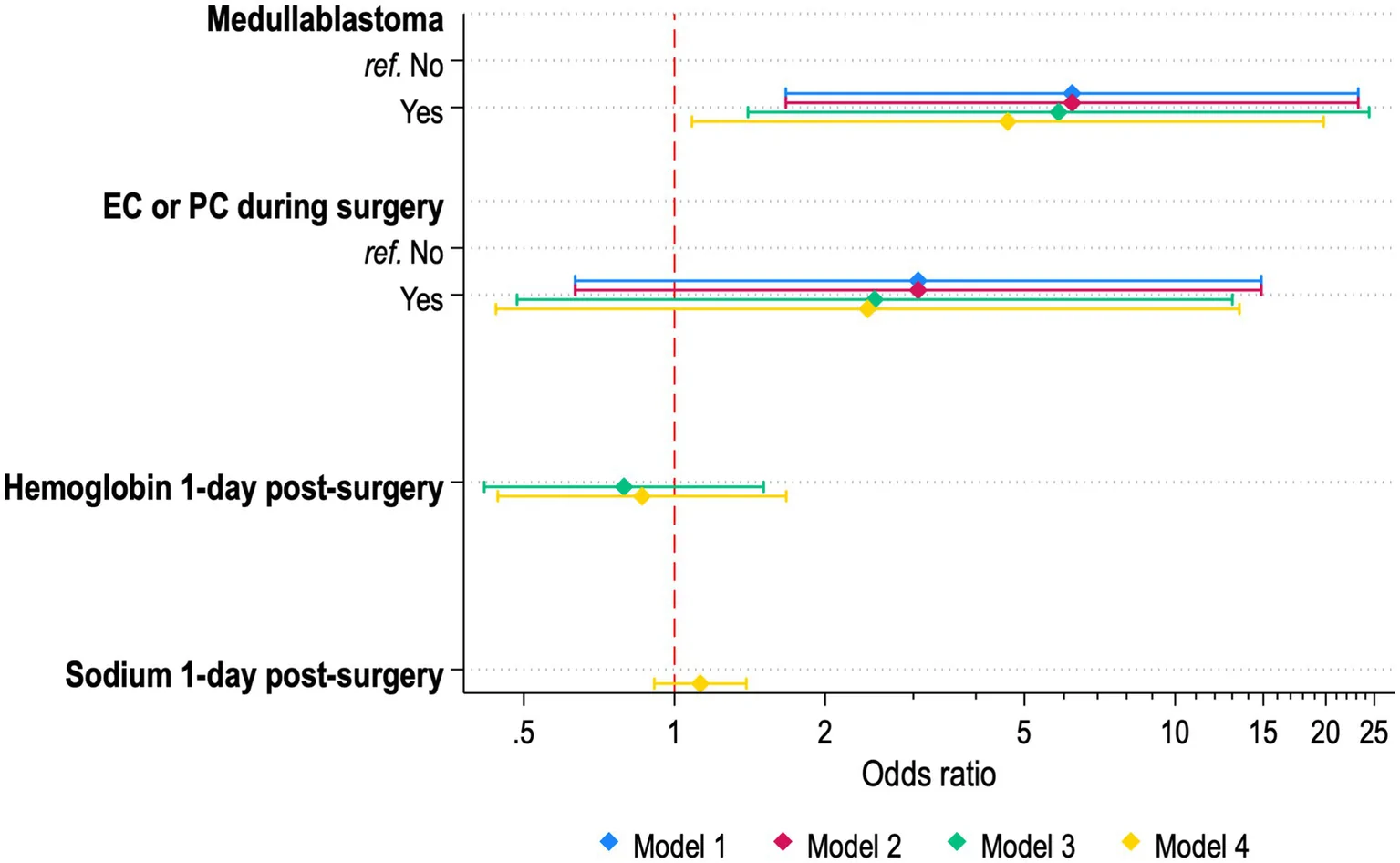
Forest plot of the multivariate analysis with different models, with the factors listed on the *y*-axis and the odds ratio on the *x*-axis.

## Discussion

The objective of this explorative study was to evaluate several potential perioperative factors that are could be critical in the development of pCMS. We identified medulloblastoma histology as an independent risk factor for developing pCMS in all models. Focusing on the perioperative factors, there were no significant associations in the multivariate analysis, although we noted some potential links in the bivariate analysis. Because the clinical manifestation of pCMS is not evident preoperatively, even in the presence of these lesion- and imaging-specific aspects, our findings are compatible with the second-hit theory. This theory proposes that disruptions may trigger neurofunctional disturbances leading to pCMS.

Consistent with this concept, further investigation into the effect of surgery as a potential second hit is warranted. Most of the existing literature emphasizes preoperative risk stratification using imaging-based tools, such as the Rotterdam Risk Score, in combination with lesion-specific aspects such as tumor histology (19).

### Lesion size and tumor characteristics

The Rotterdam Risk Score was designed to assess the anatomical and tumor-related factors preoperatively to assess the risk of developing pCMS (9). However, recent studies have reported that is has limited predictive value, consistent with our findings. Even for the assessment of compression of distinct anatomical structures or brainstem infiltration, interobserver agreement was unreliable (20). In our cohort, there were no significant differences in the score between the case and control groups. These results suggest that preoperative risk stratification based solely on lesion size and location may be insufficient to predict pCMS. The detailed anatomical location of specific tracts within the developing neuronal circuits in children does not seem to fully account for the occurrence of pCMS in individual patients (Figure 2).

**Figure 2:**
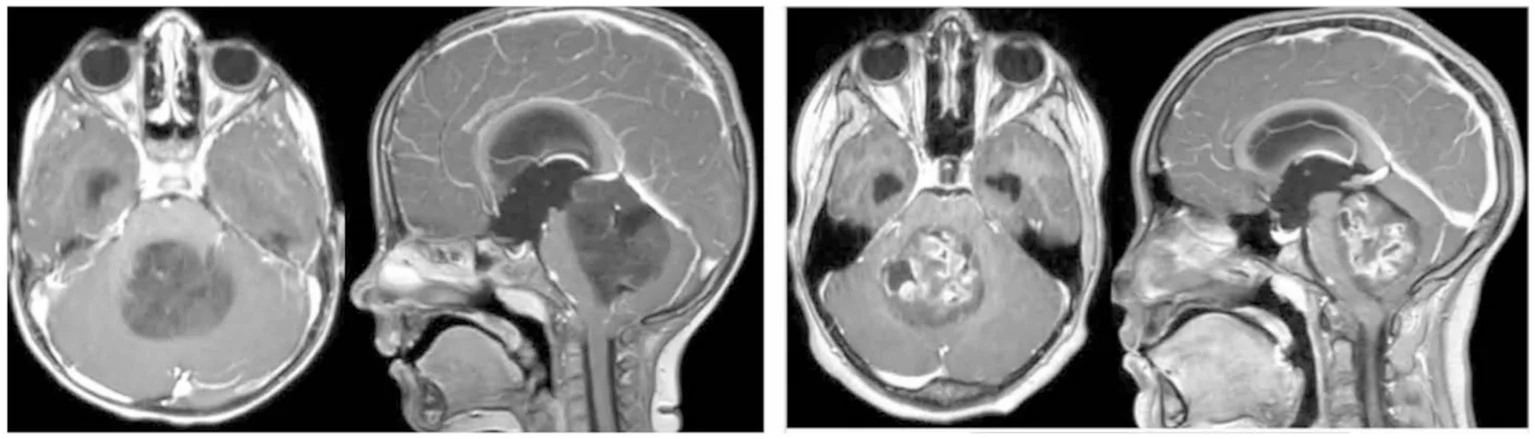
The occurrence of postoperative cerebellar mutism syndrome depending on tumor size and location. The panel on the left is from a patient with this condition, and the panel on the right is from a patient without this condition. Both patients presented with a large infratentorial lesion in a midline location as examples from the patient cohort.

### Perioperative factors

Among the perioperative factors, there were no associations in the multivariate analysis, but there were some trends in the bivariate analysis. In particular, intraoperative administration of blood products and sodium levels on postoperative day one showed patterns suggesting potential relevance, which may reach significance in larger cohorts. There were no other significant associations. These results are in line with other studies, indicating a intraoperative decrease in hematocrit and hemoglobin might be linked with the development of pCMS. Accordingly, our study and others show significant results in uni- or bivariate analysis, but with no significant effect in multivariate setting (4) suggesting a more hypothesis-generating approach. Our study showed no effect of perioperative administration of glucocorticoids on the occurrence of pCMS, consistent with a recent study that found no effect on postoperative speech impairment in general after posterior fossa tumor surgery in pediatric patients (21). These findings indicate that perioperative influences may contribute to pCMS development, but further investigation with a larger sample size and in a multicenter setting is needed.

### Neurodevelopmental considerations

The high incidence of pCMS in pediatric patients compared with adults is likely related to neurodevelopmental factors involving motor speech and language control (3, 4, 6, 10, 11). Several studies have demonstrated an age dependency in the occurrence of the pCMS. Specifically, younger patients are at a higher risk (2), and cases in patients older than 18 years are very rare and mostly presented as case reports (15). This pattern suggests that age-dependent neural circuits, particularly those governing motor speech control and language, may be more vulnerable to disruption in children (10, 15). Given this neurofunctional and neuroanatomical context, further evaluation of neurodevelopment changes as a basis for pCMS is warranted. Maturation of handedness and language networks across childhood may influence the susceptibility to injury that favors pCMS, particularly during processes such as cerebral-cerebellar lateralization. Since maturation might differ between sexes, so far no consensus could be established, whether sex is a independent factor contributing to the development of pCMS. A recent review stated that male sex might be a protective factor in a univariate analysis (22), while others reported male sex as a risk factor for developing pCMS (23).

Another recent study on connectomics involved in pCMS could provide the bridge from neuroimaging to more functional explanation for age-dependency in terms of network activity changes during development (14). Cerebello-ceberal networks do not seem to mature on the same timeline with even separate sensitive windows for injury. Frontal and motor regions thereby present a decreased connectivity with further maturation. Disruption of prefrontal cerebro-cortical connectivity during a transient period of network emergence may result in pCMS in younger children. This might explain the predominantly clinical presentation with mutism in younger children. Cognitive and social consequences of cerebellar injury show an opposite age dependence from motor consequences. Disruption of motor-related cerebellar regions are more likely to improve over time (14). In the pCMS context pediatric and adult connectomics are comprised of distinct regions namely the dorsolateral prefrontal cortex, anterior cingulate cortex, precuneus and right caudate regions. In contrast, the connectomic pattern in adults includes posterior-central regions like inferior parietal oder postcentral cortices. Red nulcei and thalami seem to play a critical role in the mediation of these maturation-dependent changes in cerebello-cerebral network connectivity (14).

### Significance of lateralization

Possible ways to assess the individual status of lateralization or maturation of cerebellar-cerebral diathesis might be handedness or language network formation, since these clinical function has been shown to be linked to lateralization processes supra- and infratentorially. So far these theoretical considerations have not been established as proven factors with the inherent difficulties to properly measure handedness and language function in young children.

### Language

Functional imaging studies using functional magnetic resonance imaging (fMRI) or single-photon emission computed tomography (SPECT) have established cerebellar involvement in motor and non-motor processes, with the right lateral posterior cerebellum connecting to the language-dominant left frontal, parietal, and temporal association areas (24). In children, the fundamental language network is established by the age of 4 years (25). Early cerebellar injury can lead to supratentorial white matter and grey matter decline (16). Gelinas et al. (26) suggested that language processing is lateralized to the right cerebellar hemisphere. Supporting this, Wibroe et al. (6) observed that postoperative speech and language difficulties occur more often after non-midline surgeries on the left compared with the right (6).

### Handedness

Left-handedness has been identified as a potential risk factor for developing pCMS (3, 5, 27). Law et al. (3) demonstrated compromised white matter in the right cerebellar region of children with pCMS, indicating that disruption of the right cerebellum and left frontal cortex may contribute to postoperative language deficits. A recent review did support the hypothesis of left-handedness being a risk factor, though this could be shown only through a univariate analysis (27). In contrast, a prospective study from the CMS group could not demonstrate a different risk developing postoperative speech impairment in relation to handedness (28).

### Intraoperative neurophysiological monitoring and pCMS

Given the proposed neurofunctional basis of pCMS and the variability in individual neuronal maturation, intraoperative monitoring may minimize injury to neurological tracts and pathways. However, monitoring cerebellar function remains challenging, and evoked potentials are not consistently obtainable in pediatric or adult patients (29, 30). Recent approaches in pediatric patients include combining local cortical cerebellar stimulation with timed transcortical motor cortex stimulation to improve reliability (29). The initial experiences with electroencephalography (EEG) examination suggest its potential for monitoring functional changes in pediatric cohorts with pCMS (31).

### Cerebello-cerebral hypoperfusion and homeostatic dysregulation

Postoperative imaging has demonstrated both bilateral frontal hypoperfusion and cerebellar hypoperfusion in patients with pCMS (18). This frontal lobe hypoperfusion in association with cerebellar efferent pathway injury could be confirmed by a recent review (32). Rare cases of non-surgical pCMS suggest a link between homeostatic disturbances and cerebellar hypoperfusion that contributes to the clinical presentation of pCMS (33). Consistently, reduced postoperative hemoglobin and hematocrit have been described in patients with pCMS, suggesting that surgically induced hypoperfusion due to bleeding may play a role (4). pCMS has also been reported in pediatric patients with acute cerebellitis in leukemia and viral illness (including COVID-19-associated cerebellitis), supporting the hypothesis that homeostatic imbalance rather than mechanical injury may contribute to the pathogenesis (33, 34).

A prospective multicenter study evaluating the effect of perioperative factors such as the administration of corticosteroids and intraoperative blood loss on pCMS is ongoing (6). In our cohort, corticosteroids did not show a significant affect, although there was a trend for hemoglobin differences between patients with pCMS and controls.

## Conclusion

Despite the relatively small sample size, our explorative study suggests that intraoperative factors may contribute to the occurrence of pCMS in children. Parameters such as administration of blood loss and transfusion requirements suggest a potential link. Therefore, careful surgical planning is necessary for posterior fossa tumors in children. Individual intraoperative neurophysiological monitoring may assist the surgeon in preserving critical pathways in the future, and the consideration of second-look surgery should be discussed in collaboration with pediatric oncology teams to achieve a maximum safe resection. Additional studies are needed to investigate either homeostatic variables and intraoperative factors to identify potentially modifiable risk contributors as well as patient’s maturation-specific contributions for preoperative planning. Protocols for intraoperative neuromonitoring beyond mere feasibility might help draw attention to the structures that must be preserved during surgery. Im total, it may be time to move beyond structural risk assessment in evaluating a highly individual, maturation-dependent disruption of network activity.

### Limitations

Standardized definitions of pCMS are lacking. The Icelandic Delphi consensus has proposed initial efforts to standardize diagnostic criteria and pre- and postoperative assessment tools. The diagnosis of pCMS remains clinical, often supplemented with a neuropsychological examination, as no imaging can definitively confirm pCMS. In this retrospective study, the diagnosis was based on pediatrician assessment without standardized questionnaires or measurements, which may have influenced incidence reporting. Studies vary in patient age groups and some variables are age dependent (e.g., handedness), or cannot be measured in very young children. We based our examination on age-matched cohorts but did not evaluate age as an independent risk factor for pCMS. Thus, we cannot directly exclude residual age-dependent effects. Furthermore, the clinical presentation of pCMS might differ with the maturation stage, and cases in early infancy may be underrecognized (2–4, 19). Due to the retrospective study design with real-world data sets from a single institution, missing values do occur during the clinical course of the individual patient. Since some laboratory parameters are highly dependent on handling technique and reference ranges may differ across laboratories, this may affect the conclusiveness of the statistical testing. These are noted as such in the tables. Furthermore a possible collinearity between the tumor size and vascularization and postoperative hemoglobin levels cannot be ruled out. Handedness has not been evaluated in the patients, nor was information on molecular subgroups for the different etiologies available for most of the included patients. Therefore, no conclusion can be drawn regarding the influence of histological subclasses on the occurrence of pCMS. More broadly, unmeasured anatomical and surgical variables – including tumor location, vermian/midline involvement, brainstem or superior cerebellar peduncle involvement, tumor vascularity, and overall surgical complexity – were not incorporated into our models and therefore represent sources of residual confounding that this analysis cannot exclude. Another limitation relates to the statistical method of forward blockwise regression, such as the risk of overfitting and variable selection instability. The main aim here was to develop an initial interpretable model to serve as a foundation for subsequent analysis. With only 20 pCMS events and four variables in the final multivariable model, the resulting events-per-variable (EPV) ratio of approximately 5 falls below the conventionally recommended threshold of 10, and estimates are inherently imprecise. Simulation studies indicate that EPV values of 5–9 can still yield acceptable estimates when candidate predictors are pre-selected on clinically and statistically justified grounds (35), as was done here – only variables significant in prior bivariate analysis were carried forward, rather than an unrestricted stepwise search among all candidates. Given the exploratory nature of this analysis, these estimates should be interpreted as hypothesis-generating rather than confirmatory. A complete-case sensitivity analysis without imputation yielded consistent findings (Supplementary Table S1). Future confirmatory studies in larger, multicentric cohorts should apply more conservative, prespecified sample-size frameworks, such as those proposed by Riley et al. (36). The general limitations of the single-center design, including only first operations and excluding revisions, must also be noted. On the other hand, this leads to the assumption of a relatively homogeneous surgical strategy across the patient cohort.

## Data Availability

The data analyzed in this study is subject to the following licenses/restrictions: the data that support the findings of this study are available from the corresponding author upon reasonable request. Requests to access these datasets should be directed to steffiott@gmx.net.
