# Supplementary material for: Association between perioperative factors and the occurrence of postoperative cerebellar mutism syndrome in children
Source: Front Neurol. 2026 Sep 23;17:1862663. doi: 10.3389/fneur.2026.1862663 (PMC13642208; doi:10.3389/fneur.2026.1862663)
Supplement: Supplementary file 1 [file Table_1.DOCX]

**Supplementary Table S1. Complete-case sensitivity analysis: associations between relevant exposure variables and post-operative pediatric CMS (no imputation, weighted by coarsened exact matching)**

|  | **Model 1** | | | **Model 2** | | | **Model 3** | | | **Model 4** | | |
| --- | --- | --- | --- | --- | --- | --- | --- | --- | --- | --- | --- | --- |
|  | **OR‡** | **95% CI Lower** | **Upper** | **OR‡** | **95% CI Lower** | **Upper** | **OR‡** | **95% CI Lower** | **Upper** | **OR‡** | **95% CI Lower** | **Upper** |
| **Medulloblastoma** | | | | | | | | | | | | |
| No | 1.00 |  |  | 1.00 |  |  | 1.00 |  |  | 1.00 |  |  |
| Yes | **7.86** | 2.19 | 28.17 | **6.22** | 1.67 | 23.21 | **5.39** | 1.28 | 22.73 | **4.63** | 1.08 | 19.79 |
| **EC or PC during surgery** | | | | | | | | | | | | |
| No |  |  |  | 1.00 |  |  | 1.00 |  |  | 1.00 |  |  |
| Yes |  |  |  | 3.07 | 0.63 | 14.86 | 2.44 | 0.47 | 12.66 | 2.43 | 0.44 | 13.44 |
| **Hemoglobin, day 1 post-surgery (g/dL)** | | | | | | | | | | | | |
|  |  |  |  |  |  |  | 0.75 | 0.39 | 1.46 | 0.86 | 0.44 | 1.67 |
| **Sodium, day 1 post-surgery (mmol/L)** | | | | | | | | | | | | |
|  |  |  |  |  |  |  |  |  |  | 1.13 | 0.91 | 1.39 |
| **N** | | | | | | | | | | | | |
|  | 69 | | | 68 | | | 62 | | | 61 | | |

*OR, odds ratio; CI, confidence interval; EC, erythrocyte concentrates; PC, platelet concentrates; mmol/L, millimoles per liter; g/dL, gram per deciliter.*

*‡ Adjusted for the matching variable (age at diagnosis, months) and sex, weighted by coarsened exact matching weights. Complete-case analysis (listwise deletion); N decreases across models 1-4 due to missing values in the added predictors. Compare with Table 2 (multiple imputation, m=30), which uses the same matched analytic sample (N=69) throughout.*

*Estimates are highly consistent between the complete-case and multiple-imputation analyses, supporting the robustness of the medulloblastoma association in particular.*
